# Racial Disparities in MiT Family Translocation Renal Cell Carcinoma

**DOI:** 10.1093/oncolo/oyad173

**Published:** 2023-06-14

**Authors:** Xiaofan Lu, Nassim Tawanaie Pour Sedehi, Xiaoping Su, Fangrong Yan, Omar Alhalabi, Nizar M Tannir, Gabriel G Malouf

**Affiliations:** Department of Cancer and Functional Genomics, Institute of Genetics and Molecular and Cellular Biology, CNRS/INSERM/UNISTRA, Illkirch, France; Department of Cancer and Functional Genomics, Institute of Genetics and Molecular and Cellular Biology, CNRS/INSERM/UNISTRA, Illkirch, France; Department of Bioinformatics and Computational Biology, The University of Texas MD Anderson Cancer Center, Houston, TX, USA; Research Center of Biostatistics and Computational Pharmacy, China Pharmaceutical University, Nanjing, People’s Republic of China; Department of Genitourinary Medical Oncology, The University of Texas MD Anderson Cancer Center, Houston, TX, USA; Department of Genitourinary Medical Oncology, The University of Texas MD Anderson Cancer Center, Houston, TX, USA; Department of Cancer and Functional Genomics, Institute of Genetics and Molecular and Cellular Biology, CNRS/INSERM/UNISTRA, Illkirch, France; Department of Medical Oncology, Institut de Cancérologie de Strasbourg, Strasbourg University, Strasbourg, France

**Keywords:** *TFE3*, *TFEB*, translocation renal cell carcinomas, race

## Abstract

Racial disparities have been documented in the biology and outcome of certain renal cell carcinomas (RCCs) among Black patients. However, little is known about racial differences in MiT family translocation RCC (TRCC). To investigate this issue, we performed a case-control study using data from The Cancer Genome Atlas (TCGA) and the Chinese OrigiMed2020 cohort. A total of 676 patients with RCC (14 Asian, 113 Black, and 525 White) were identified in TCGA, and TRCC was defined as RCC with *TFE3*/*TFEB* translocation or *TFEB* amplification, leading to 21 patients with TRCC (2 Asian, 8 Black, 10 White, and 1 unknown). Asian (2 of 14 [14.3%] vs 10 of 525 [1.9%]; *P* = .036) and Black (8 of 113 [7.1%] vs 1.9%; *P* = .007) patients with RCC showed significantly higher prevalence of TRCC compared with White patients with RCC. The overall mortality rate of TRCC was slightly higher in Asian and Black patients compared with White patients (HR: 6.05, *P* = .069). OrigiMed2020 Chinese patients with RCC had a significantly higher proportion of TRCC with *TFE3* fusions than TCGA White patients with RCC (13 of 250 [5.2%] vs 7 of 525 [1.3%]; *P* = .003). Black patients with TRCC were more likely to exhibit the proliferative subtype than White patients (6 of 8 [75%] vs 2 of 9 [22.2%]; *P* = .057) for those who had RNA-seq profiles. We present evidence of higher prevalence of TRCC in Asian and Black patients with RCC compared with White patients and show that these tumors in Asian and Black patients have distinct transcriptional signatures and are associated with poor outcomes.

## Teaser

Little is known about racial differences in MiT family translocation renal cell carcinomas (TRCC). This study investigated the issue using data from The Cancer Genome Atlas (TCGA) and the Chinese OrigiMed2020 cohort.

## Introduction

Renal cell carcinoma (RCC) represents about 3% of all cancers, with varying incidence and mortality worldwide.^1,2^ The most common histology of RCC is clear cell (ccRCC), followed by 20 distinct histopathological entities such as papillary RCC (PRCC) among others.^3^ Studies have shown inferior overall survival in Black patients with RCC compared with White patients with RCC.^4-6^ It was recently found that differences in germline variants exist among patients with RCC of different ancestries. Approximately 17% of patients with RCC carry pathogenic or likely pathogenic variants, with *FH* variants being more common in patients of African ancestry, as compared with *CHEK2* variants, which are more common in patients of non-African ancestry.^7^ Even for ccRCC, several studies identified lower rates of *VHL* inactivation in Black patients, associated with lower levels of HIF and VEGF pathway alterations.^5,7,8^ MiT family translocation RCC (TRCC) is a rare and aggressive subtype of RCC that accounts for approximately 5% of all RCCs and 15% of RCCs in patients under 40 years of age.^9^ In the last World Health Organization classification, RCCs with alterations in *TFE3* and *TFEB* transcription factors are classified as either *TFE3* translocation RCC or *TFEB* rearranged RCC, the latter presenting with either translocations or amplifications.^3^ Therefore, RCC subtypes characterized by alterations in MiT family genes, which share similar presentations, can be grouped together for simplicity as MiT family-altered RCC. TRCC usually has limited response to VEGFR tyrosine kinase inhibitors and immune checkpoint inhibitors, with a median progression-free survival (PFS) of 3 months.^10-13^ More effective therapies grounded in biological insights are thus needed. Except for chemotherapy during childhood, no risk factor is known to be associated with TRCC.^14^ Herein, we present an analysis of potential associations between TRCC and race, which could lead to improved management and outcomes for patients with these rare RCC subtypes.

## Methods

### Analysis of The Cancer Genome Atlas and Validation Cohorts

Genomic and clinical data for a total of 795 primary tumors from TCGA-KIRC (*n* = 512) and TCGA-KIRP (*n* = 283) cohorts were downloaded from cBioPortal (https://www.cbioportal.org/). Survival information were retrieved from cBioPortal, including PFS which indicates whether patient’s disease has recurred/progressed, and at what time the disease recurred or the patient was last seen, and disease-free survival (DFS) which refers to disease-free time since initial treatment. Of these 795 samples, 676 were profiled for both structural and copy number variation, and 17 cases were detected with *TFE3* or *TFEB* translocations. As described in our previous report,^15^ one case (TCGA-A3-3313-01) with *TFEB* amplification was also identified as harboring *TFEB* translocation, along with 3 PRCC cases with *TFEB* amplification. An independent validation cohort, the Chinese pan-cancer cohort (OrigiMed2020), was also investigated.^16^ Participants’ race and ethnicity and the source of the classifications were identified by self-report or selection, or by electronic heath record if necessary for both cohorts. For the purpose of this study, all MiT family-altered RCCs were referred to as TRCC.

Transcriptome expression profiles for TCGA cohort were retrieved and analyzed using transcripts per kilobase million. The potential cross-cohort batch effect was removed using an empirical Bayesian framework,^17^ and principal component analysis was used to further investigate this effect. We selected genes that were uniquely upregulated for each of the 7 subtypes previously described (log_2_FoldChange > 1 and adjusted *P* < .05).^18^ Each TRCC case was classified as one of these 7 subtypes through nearest template prediction.^19^

### Statistical Analyses

All statistical tests were executed by R (v4.0.2), including Fisher’s exact test for categorical data with odds ratio (OR) and 95% confidence interval (CI), 2-sample Mann-Whitney *U* test for continuous data, log-rank test with Kaplan-Meier curves, and Cox proportional hazards regression for hazard ratio (HR) with 95% CI. For unadjusted comparisons, a 2-sided *P* < .05 was considered statistically significant.

## Results

**Figure 1.**
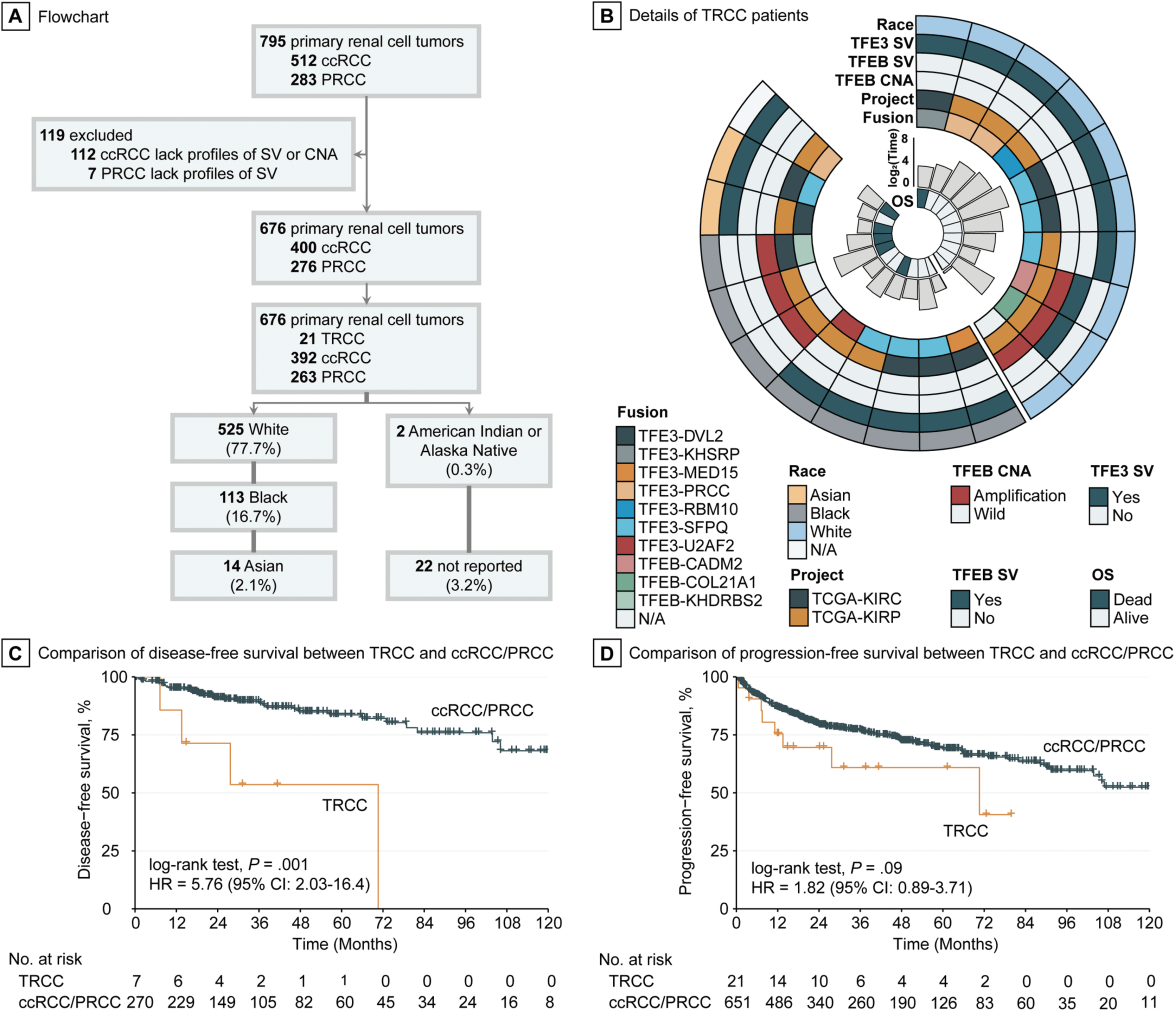
Identification of TRCC in TCGA cohort and its prognostic relevance. (**A**) Inclusion criteria for TCGA cohort to investigate racial disparity in TRCC. (**B**) Landscape of race, translocation/amplification, and clinical outcome of TRCC in TCGA cohort. (**C**) Disease-free survival curve of TRCC versus ccRCC/PRCC. (**D**) Progression-free survival curve of TRCC versus ccRCC/PRCC.

Among the 676 patients with RCC from TCGA cohort, 2 (0.3%) were American Indian or Alaska Native, 14 (2.1%) Asian, 113 (16.7%) Black, and 525 (77.7%) White; for 22 (3.2%) patients, race was not reported (Fig. 1A). We identified a total of 21 patients with RCC (2 Asian, 8 Black, and 10 White patients) who had *TFE3*/*TFEB* translocations or *TFEB* amplification (Fig. 1B; Supplementary Table S1). We found that patients with TRCC had significantly shorter DFS compared with patients with RCC who did not have TRCC (median DFS: 70.6 months vs “not reached,” *P* = .0002; Fig. 1C). A trend toward shorter PFS was also observed in patients with TRCC compared with other patients with RCC (median PFS: 70.6 vs 123.8 months, *P* = .09; Fig. 1D). Patients with TRCC had significantly higher likelihood of lymph node involvement (*P* = .001) and tended to have higher T stage (*P* = .099) and pathological stage (*P* = .089). In addition, patients with TRCC harbored lower tumor mutation burden (*P* = .04) and aneuploidy scores (*P* = .07) (Supplementary Table S2). When considering samples with common mutations occurring in at least 5% of cases, only one single TRCC case harbored both *VHL* and *PBRM1* mutations, while over 40% of ccRCCs displayed these mutations. Furthermore, TRCC did not exhibit any mutations in *SETD2* and *BAP1*, which were observed in more than 10% of ccRCCs (Supplementary Table S3). Notably, there were no significant differences in the mutation frequency of common genes between TRCC and PRCC (Supplementary Table S3).

**Figure 2.**
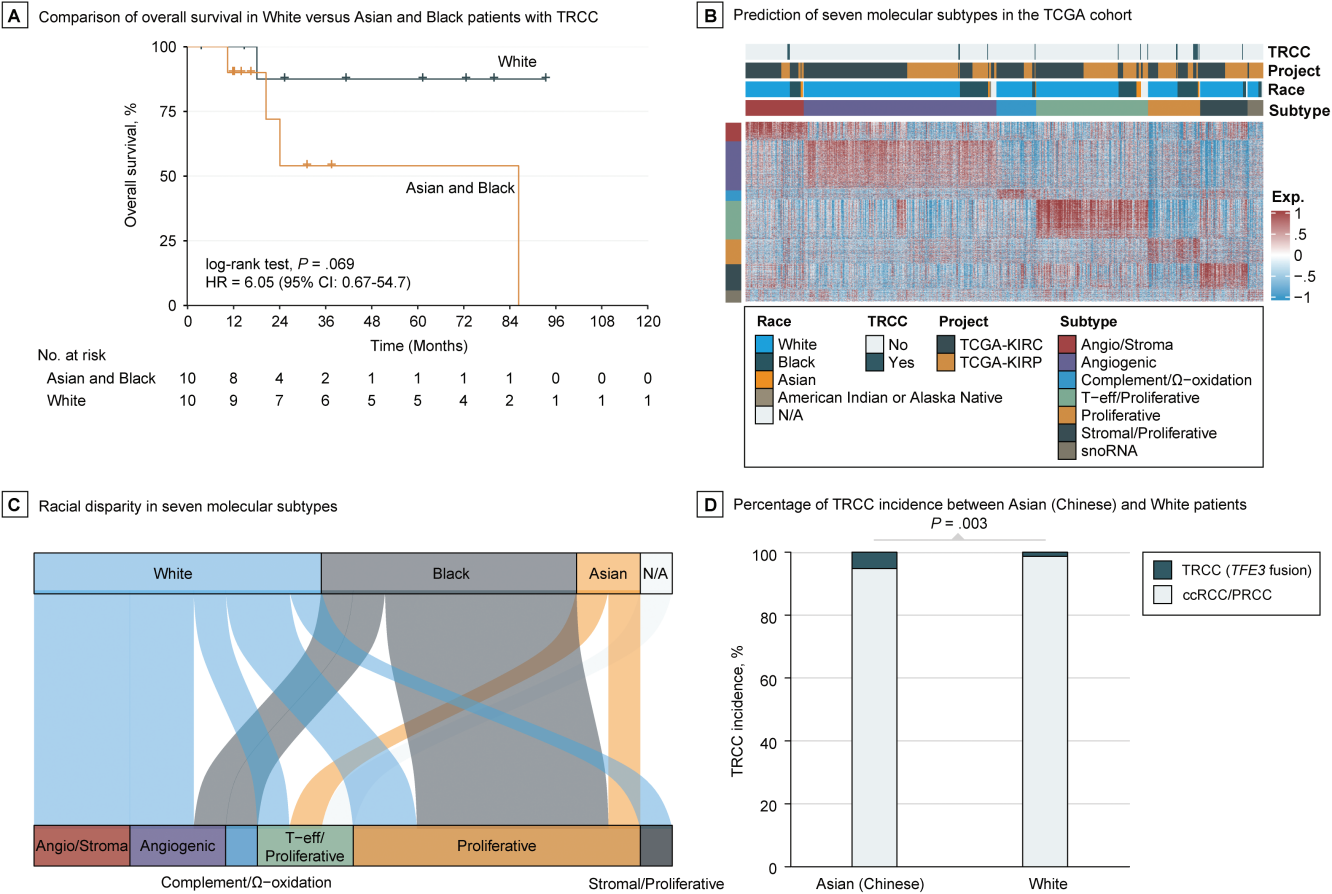
Racial disparity in the incidence and clinical outcome of TRCC. (**A**) Overall survival curve White versus Asian and Black patients with TRCC. (**B**) Heatmap of transcriptional landscape of TCGA cohort by nearest template prediction using signature that was derived from Genentech’s profiling performed on tumors from patients enrolled on IMmotion151. (**C**) Association between race and predicted molecular subtypes in TRCC from TCGA cohort. (**D**) Incidence rate of TRCC between Chinese and White populations using Chinese OrigiMed2020 and TCGA cohorts.

We found that Black patients had significantly higher prevalence of TRCC compared with White patients (7.1% vs 1.9%; OR: 3.9, 95% CI: 1.3-11.3, *P* = .007). Likewise, Asian patients also had a significantly higher proportion of TRCC compared with White patients (14.3% vs 1.9%; OR: 8.5, 95% CI: 0.8-46.9, *P* = .036). The overall mortality rate of TRCC marginally increased in Asian and Black patients compared with White patients (HR: 6.05, 95% CI: 0.67-54.7, *P* = .069; Fig. 2A); race might be an independent prognostic factor in TRCC when adjusting for age and pathological stage (HR: 8.91, 95% CI: 0.87-91.01, *P* = .065; Supplementary Table S4). However, no statistical differences were observed in major clinicopathological and molecular features (all *P* > .3; Supplementary Table S5), nor in genetic mutations (all *P* > .2; data not shown), in Asian and Black vs White patients with TRCC. Among the 18 cases with detected fusions, there was no statistical association between *TFE3* and *TFEB* fusion partners and race (*P* = .697). Of the 20 TRCC cases with available RNA sequencing profiles, nearly half (45%) were predicted to be of the proliferative subtype (Fig. 2B; Supplementary Fig. S1 and Supplementary Table S6). Black patients with TRCC were more likely than White patients with TRCC to exhibit the proliferative transcriptomic subtype (75% vs 22.2%, *P* = .057; Fig. 2C).

The Chinese OrigiMed2020 cohort included 308 patients with RCC belonging to 6 phenotypes (Supplementary Fig. S2). Of these, 32 patients (10.4%) had *TFE3* translocations, with no *TFEB* fusions or amplifications reported. Of these 32 patients, 22 were initially diagnosed with TRCC by pathologists, 13 with ccRCC, and 2 unclassified. Therefore, 13 out of 233 (5.6%) ccRCC cases were misclassified. This rate is similar to the misclassification rates observed in TCGA-KIRC (2%) and -KIRP cohorts (4.7%). To compare the incidence rate of TRCC between Chinese and White populations, we focused on TRCC cases misclassified as ccRCC (*n* = 233) or PRCC (*n* = 17) in the OrigiMed2020 cohort, which allowed a comparative scenario with TCGA cohort. Notably, *TFEB* amplifications and fusions were not included in the OrigiMed2020 cohort due to the nature of the targeted assay used; thus, only TRCC cases with *TFE3* fusions were considered. Overall, 13 out of 250 Chinese patients with RCC in the OrigiMed2020 cohort had *TFE3* translocations, while only 7 out of 525 White patients with RCC in TCGA cohort had *TFE3* translocations (5.2% vs 1.3%; OR: 4.1, 95% CI: 1.5-12.2, *P* = .003; Fig. 2D). These data suggest that racial disparities exist in the development of TRCC, with White patients being less likely to develop TRCC compared with Asian and Black patients.

## Discussion

To our knowledge, this is the first report to examine racial differences in TRCC between Asian and Black versus White patients. Our data indicate a higher incidence of TRCC in Asian and Black patients compared with White patients, and no difference in fusion partners in patients with TRCC among these 3 races. Transcriptomics show a proliferative gene expression signature in 75% of Asian and Black patients and 22.2% of White patients with TRCC using Genentech’s profiling performed on tumors from patients enrolled on IMmotion151. This signature has been associated with poor-risk groups of metastatic ccRCC; these patients have shown resistance to sunitinib, but an improved objective response rate and PFS when treated with atezolizumab and bevacizumab.^18^ These findings point toward the potential role of host factors or germline genomic variations in TRCC development, although other factors cannot be ruled out. Therefore, we postulate that Asian and Black patients with RCC should undergo additional molecular testing to accurately diagnose TRCC and avoid misdiagnosis as ccRCC or PRCC.

Finally, why Asian and Black patients have higher prevalence of TRCC remains to be elucidated. One possibility is that the development of TRCC in Asian populations might be related to exposure to aristolochic acid, a type of carcinogen from traditional Chinese herbs, as recently demonstrated in a large study analyzing the mutational signature of Asian patients with TRCC.^20^ Another possibility is related to differences in the biological underpinnings of RCC in African versus European populations, as was recently demonstrated.^7^ We acknowledge several limitations in our study. Firstly, the small number of TRCC cases represents a limitation, which is consistent with the rarity of this subtype. Secondly, it is worth noting that the pathological images for these cases might not have undergone centralized review, introducing potential variations in assessment. Thirdly, the unavailability of treatment information could introduce bias in the survival analyses. Larger epidemiological studies are needed to shed light on the contributions of race, host exposures, and RCC subtypes.

## Conclusions

Despite improvements in understanding the biology of TRCC, the origin of the racial disparity in the development of these tumors remains unclear. We present evidence of higher prevalence of TRCC in Asian and Black patients compared with White patients and show that these tumors in Asian and Black patients have distinct transcriptional signatures and are associated with poor outcomes. While it is plausible that genetic variation might explain these differences, we could not exclude the contribution of exposure to carcinogens.

## Supplementary Material

Supplementary material is available at *The Oncologist* online.

oyad173_suppl_Supplementary_Figure_S1

oyad173_suppl_Supplementary_Figure_S2

oyad173_suppl_Supplementary_Table_S1

oyad173_suppl_Supplementary_Table_S2

oyad173_suppl_Supplementary_Table_S3

oyad173_suppl_Supplementary_Table_S4

oyad173_suppl_Supplementary_Table_S5

oyad173_suppl_Supplementary_Table_S6

## Data Availability

The public data analyzed in this study were obtained from cBioPortal for TCGA-KIRC, TCGA-KIRP, and Chinese OrigiMed2020 datasets (https://www.cbioportal.org/). The data regarding details of the 21 TRCC cases identified in the TCGA cohort are available in Supplementary Material.
