# Supplementary material for: Racial Disparities in MiT Family Translocation Renal Cell Carcinoma
Source: Oncologist. 2023 Jun 14;28(11):1009–13. doi: 10.1093/oncolo/oyad173 (PMC10628562; doi:10.1093/oncolo/oyad173)
Supplement: oyad173_suppl_Supplementary_Figure_S1 [file oyad173_suppl_supplementary_figure_s1.docx]

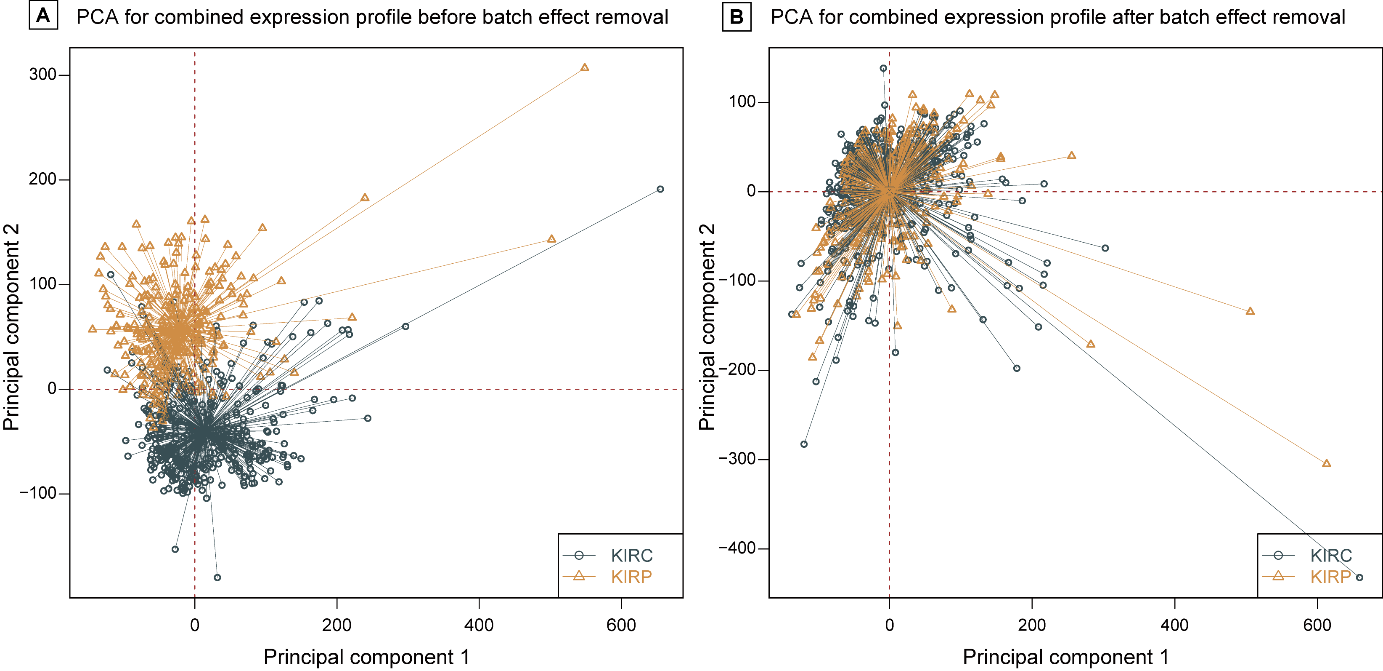


**Figure S1. Batch effect across the TCGA-KIRC and -KIRP cohorts. (A)** Principal component analysis for combined expression profile of two cohorts before removal of batch effect. **(B)** Principal component analysis for combined expression profile of two cohorts after removal of batch effect.
