# Supplementary material for: Racial Disparities in MiT Family Translocation Renal Cell Carcinoma
Source: Oncologist. 2023 Jun 14;28(11):1009–13. doi: 10.1093/oncolo/oyad173 (PMC10628562; doi:10.1093/oncolo/oyad173)
Supplement: oyad173_suppl_Supplementary_Figure_S2 [file oyad173_suppl_supplementary_figure_s2.docx]

**
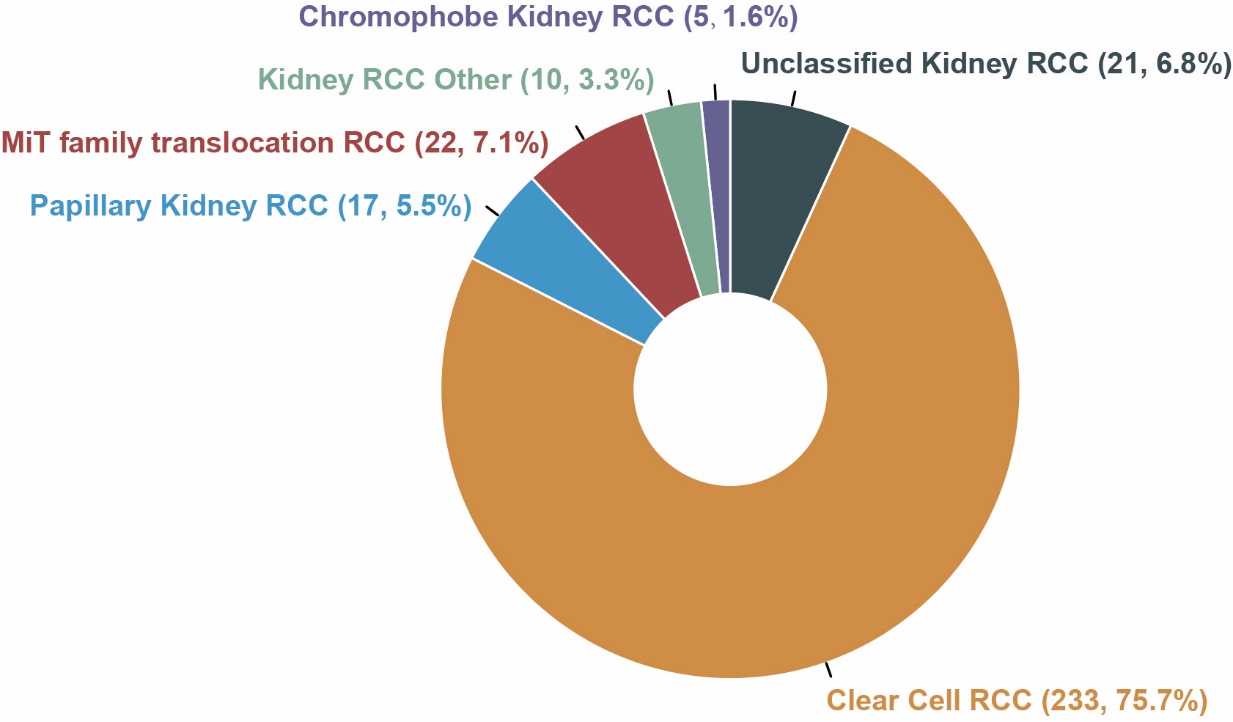
**

**Figure S2. Categories of renal cell carcinoma in Chinese OrigiMed2020 cohort.** A total of 308 Chinese renal cell carcinoma (RCC) patients that categorized in six phenotypes were investigated in the validation Chinese OrigiMed2020 cohort.
