# Supplementary material for: Racial Disparities in MiT Family Translocation Renal Cell Carcinoma
Source: Oncologist. 2023 Jun 14;28(11):1009–13. doi: 10.1093/oncolo/oyad173 (PMC10628562; doi:10.1093/oncolo/oyad173)
Supplement: oyad173_suppl_Supplementary_Table_S1 [file oyad173_suppl_supplementary_table_s1.docx]

**Table S1.** Details of 21 patients with TRCC identified in the TCGA cohort

| **SampleID** | **Race** | **SV** | **CNA (Amp)** | **Fusion partner** | **Subtype** |
| --- | --- | --- | --- | --- | --- |
| KIRC-5681-01 | White | TFE3 |  | KHSRP | Proliferative |
| KIRP-5887-01 | White | TFE3 |  | PRCC | Angio/Stroma |
| KIRP-7050-01 | White | TFE3 |  | PRCC | Proliferative |
| KIRP-6131-01* | White | TFE3 |  | RBM10 |  |
| KIRC-5705-01 | White | TFE3 |  | SFPQ | Angiogenic |
| KIRC-4758-01 | White | TFE3 |  | SFPQ | T-eff/Proliferative |
| KIRP-A7SO-01 | White | TFE3 |  | SFPQ | Angiogenic |
| KIRP-A69E-01 | White | TFEB | TFEB | CADM2 | Angio/Stroma |
| KIRP-7048-01 | White | TFEB | TFEB | COL21A1 | Angio/Stroma |
| KIRP-7966-01 | White |  | TFEB |  | Stromal/Proliferative |
| KIRC-AA2E-01 | African American | TFE3 |  | MED15 | Proliferative |
| KIRC-3456-01 | African American | TFE3 |  | SFPQ | Proliferative |
| KIRC-5546-01 | African American | TFE3 |  | SFPQ | Proliferative |
| KIRP-7501-01 | African American | TFE3 |  | SFPQ | Proliferative |
| KIRP-A9JO-01 | African American | TFE3 |  | U2AF2 | Proliferative |
| KIRP-A5QZ-01 | African American |  | TFEB |  | Complement/Ω-oxidation |
| KIRP-A9PQ-01 | African American |  | TFEB |  | Angiogenic |
| KIRC-3313-01 | African American | TFEB | TFEB | KHDRBS2 | Proliferative |
| KIRP-8537-01 | Asian | TFE3 |  | DVL2 | T-eff/Proliferative |
| KIRC-4756-01 | Asian | TFE3 |  | SFPQ | Proliferative |
| KIRP-5882-01 | N/A | TFE3 |  | PRCC | T-eff/Proliferative |
| Abbreviations: structural variant (SV); copy number alteration (CNA) | | | | | |
| *RNA sequencing profile is not available | | | | | |
