# Supplementary material for: Racial Disparities in MiT Family Translocation Renal Cell Carcinoma
Source: Oncologist. 2023 Jun 14;28(11):1009–13. doi: 10.1093/oncolo/oyad173 (PMC10628562; doi:10.1093/oncolo/oyad173)
Supplement: oyad173_suppl_Supplementary_Table_S2 [file oyad173_suppl_supplementary_table_s2.docx]

**Table S2.** Demographic and molecular comparison between TRCC and ccRCC/PRCC

| Variables | TRCC | ccRCC/PRCC | *P*-value |
| --- | --- | --- | --- |
|  | (n = 21) | (n = 655) |  |
| Age | 55 [44, 62] | 61 [52, 70] | 0.011 |
| Sex |  |  | 0.058 |
| Female | 11 (52.4) | 208 (31.8) |  |
| Male | 10 (47.6) | 447 (68.2) |  |
| Race |  |  | 0.003 |
| American Indian or Alaska Native | 0 (0.0) | 2 (0.3) |  |
| Asian | 2 (10.0) | 12 (1.9) |  |
| Black | 8 (40.0) | 105 (16.6) |  |
| White | 10 (50.0) | 515 (81.2) |  |
| T stage |  |  | 0.089 |
| T1 + T2 | 11 (52.4) | 462 (70.8) |  |
| T3 + T4 | 10 (47.6) | 191 (29.2) |  |
| M stage |  |  | 0.703 |
| M0 | 12 (92.3) | 388 (85.3) |  |
| M1 | 1 (7.7) | 67 (14.7) |  |
| Lymph node presentation |  |  | 0.001 |
| No | 9 (45.0) | 509 (78.8) |  |
| Yes | 11 (55.0) | 137 (21.2) |  |
| Pathological stage |  |  | 0.099 |
| I + II | 10 (47.6) | 419 (66.6) |  |
| III + IV | 11 (52.4) | 210 (33.4) |  |
| Histology grade |  |  | 0.475 |
| G1 + G2 | 5 (62.5) | 171 (44.4) |  |
| G3 + G4 | 3 (37.5) | 214 (55.6) |  |
| Aneuploidy score | 2.5 [0.75, 9.5] | 6.0 [2.0, 10.0] | 0.070 |
| TMB (nonsynonymous) | 1.17 [0.8, 2.27] | 1.87 [1.27, 2.53] | 0.040 |
| *Continuous variables were presented as median (interquartile range [IQR]) and were compared using Mann-Whitney U test, while categorical variables were presented as frequency (%) and were compared using Fisher’s exact test; missing records were neither included in the frequency calculation nor in statistical inference. Abbreviation: TMB for tumor mutation burden. | | | |
