# Supplementary material for: Racial Disparities in MiT Family Translocation Renal Cell Carcinoma
Source: Oncologist. 2023 Jun 14;28(11):1009–13. doi: 10.1093/oncolo/oyad173 (PMC10628562; doi:10.1093/oncolo/oyad173)
Supplement: oyad173_suppl_Supplementary_Table_S3 [file oyad173_suppl_supplementary_table_s3.docx]

**Table S3. Comparison of somatic mutation frequency between TRCC and ccRCC/PRCC in the TCGA cohort**

| **Gene (Mutated)** | **No. of total mutation** | **TRCC** | **ccRCC/PRCC** | ***P* value** |
| --- | --- | --- | --- | --- |
| TRCC vs ccRCC |  |  |  |  |
| *VHL* | 166 (45%) | 1 (5.6%) | 165 (47.4%) | 0.0003 |
| *PBRM1* | 144 (39%) | 1 (5.6%) | 143 (41.1%) | 0.002 |
| *BAP1* | 38 (10%) | 0 | 38 (10.9%) | 0.236 |
| *SETD2* | 48 (13%) | 0 | 48 (13.8%) | 0.195 |
|  |  |  |  |  |
| TRCC vs PRCC |  |  |  |  |
| *MET* | 22 (8%) | 1 (5.6%) | 21 (8.0%) | 1 |
| *SETD2* | 17 (6%) | 0 | 17 (6.5%) | 0.612 |
| *NF2* | 9 (3%) | 1 (5.6%) | 8 (3.1%) | 0.455 |
| *KDM6A* | 11 (4%) | 0 | 11 (4.2%) | 1 |
| *SMARCB1* | 6 (2%) | 1 (5.6%) | 5 (1.9%) | 0.331 |
