# Supplementary material for: Racial Disparities in MiT Family Translocation Renal Cell Carcinoma
Source: Oncologist. 2023 Jun 14;28(11):1009–13. doi: 10.1093/oncolo/oyad173 (PMC10628562; doi:10.1093/oncolo/oyad173)
Supplement: oyad173_suppl_Supplementary_Table_S4 [file oyad173_suppl_supplementary_table_s4.docx]

**Table S4.** Summary of multivariate analysis integrating race with other features

| **Variables** | **No. of cases** | **No. of deaths (%)** | **HR (95% CI)** | ***P* value** |
| --- | --- | --- | --- | --- |
| Race |  |  |  | 0.065 |
| White (*ref.*) | 10 | 1 (10) | - |  |
| Asian and Black | 10 | 4 (40) | 8.91 (0.87-91.01) |  |
| Age (years) |  |  |  | 0.220 |
| ≤55.5 (*ref.*) | 10 | 2 (20) | - |  |
| >55 | 10 | 3 (30) | 4.45 (0.41-48.28) |  |
| Pathological stage |  |  |  | 0.180 |
| I + II (*ref.*) | 10 | 1 (10) | - |  |
| III + IV | 10 | 4 (40) | 4.94 (0.48-50.99) |  |
