# Supplementary material for: Racial Disparities in MiT Family Translocation Renal Cell Carcinoma
Source: Oncologist. 2023 Jun 14;28(11):1009–13. doi: 10.1093/oncolo/oyad173 (PMC10628562; doi:10.1093/oncolo/oyad173)
Supplement: oyad173_suppl_Supplementary_Table_S5 [file oyad173_suppl_supplementary_table_s5.docx]

**Table S5.** Demographic and molecular comparison in White versus Asian and Black patients with TRCC

| Variables | White | Asian and Black | *P*-value |
| --- | --- | --- | --- |
|  | (n = 10) | (n = 10) |  |
| Age | 54 [41, 64] | 58 [50, 61] | 0.820 |
| Sex |  |  | 0.370 |
| Female | 4 (40) | 7 (70) |  |
| Male | 6 (60) | 3 (30) |  |
| T stage |  |  | 0.370 |
| T1 + T2 | 4 (40) | 7 (70) |  |
| T3 + T4 | 6 (60) | 3 (30) |  |
| M stage |  |  | 1.000 |
| M0 | 6 (85.7) | 5 (100) |  |
| M1 | 1 (14.3) | 0 (0) |  |
| Lymph node presentation |  |  | 1.000 |
| No | 5 (50) | 4 (45.0) |  |
| Yes | 5 (50) | 5 (55.0) |  |
| Pathological stage |  |  | 0.656 |
| I + II | 4 (40) | 6 (60) |  |
| III + IV | 6 (60) | 4 (40) |  |
| Histology grade |  |  | 1.000 |
| G1 + G2 | 2 (66.7) | 3 (60) |  |
| G3 + G4 | 1 (33.5) | 2 (40) |  |
| Aneuploidy score | 2 [1.3, 3] | 7 [0, 9] | 0.967 |
| TMB (nonsynonymous) | 1.67 [0.8, 2.5] | 0.95 [0.8, 1.2] | 0.426 |
| *Continuous variables were presented as median (interquartile range [IQR]) and were compared using Mann-Whitney U test, while categorical variables were presented as frequency (%) and were compared using Fisher’s exact test; missing records were neither included in the frequency calculation nor in statistical inference. Abbreviation: TMB for tumor mutation burden. | | | |
