# Supplementary material for: Racial Disparities in MiT Family Translocation Renal Cell Carcinoma
Source: Oncologist. 2023 Jun 14;28(11):1009–13. doi: 10.1093/oncolo/oyad173 (PMC10628562; doi:10.1093/oncolo/oyad173)
Supplement: oyad173_suppl_Supplementary_Table_S6 [file oyad173_suppl_supplementary_table_s6.docx]

**Table S6.** Prediction of subtypes in 20 TRCC patients with available RNA sequencing profiles from the TCGA cohort

| **SampleID** | **Subtype^†^** | **Nearest template prediction**^§^ | | | | | | | | |
| --- | --- | --- | --- | --- | --- | --- | --- | --- | --- | --- |
|  |  | **d.NMF1** | **d.NMF2** | **d.NMF3** | **d.NMF4** | **d.NMF5** | **d.NMF6** | **d.NMF7** | ***P*-value** | **FDR** |
| KIRC-5681-01 | Proliferative | 0.7531 | 0.7616 | 0.7131 | 0.7793 | 0.5741 | 0.6913 | 0.7278 | 0.001 | 0.001 |
| KIRP-5887-01 | Angio/Stroma | 0.5944 | 0.8148 | 0.6932 | 0.6995 | 0.6671 | 0.6367 | 0.7289 | 0.001 | 0.001 |
| KIRP-7050-01 | Proliferative | 0.7250 | 0.6185 | 0.7342 | 0.7518 | 0.6152 | 0.7504 | 0.7009 | 0.001 | 0.001 |
| KIRC-5705-01 | Angiogenic | 0.7206 | 0.4779 | 0.7366 | 0.7073 | 0.7368 | 0.7785 | 0.7370 | 0.001 | 0.001 |
| KIRC-4758-01 | T-eff/Proliferative | 0.6537 | 0.6472 | 0.7838 | 0.6065 | 0.7525 | 0.7483 | 0.7033 | 0.001 | 0.001 |
| KIRP-A7SO-01 | Angiogenic | 0.7014 | 0.5855 | 0.7248 | 0.7780 | 0.6625 | 0.7612 | 0.6994 | 0.001 | 0.001 |
| KIRP-A69E-01 | Angio/Stroma | 0.5802 | 0.7045 | 0.7148 | 0.5892 | 0.6663 | 0.6942 | 0.7080 | 0.001 | 0.001 |
| KIRP-7048-01 | Angio/Stroma | 0.6186 | 0.7176 | 0.7175 | 0.7163 | 0.6762 | 0.7049 | 0.7267 | 0.001 | 0.001 |
| KIRP-7966-01 | Stromal/Proliferative | 0.6514 | 0.8334 | 0.6906 | 0.7394 | 0.6772 | 0.5884 | 0.6864 | 0.001 | 0.001 |
| KIRC-AA2E-01 | Proliferative | 0.7575 | 0.7311 | 0.7293 | 0.7546 | 0.5517 | 0.7613 | 0.7096 | 0.001 | 0.001 |
| KIRC-3456-01 | Proliferative | 0.8109 | 0.6970 | 0.7367 | 0.7493 | 0.5243 | 0.7469 | 0.7143 | 0.001 | 0.001 |
| KIRC-5546-01 | Proliferative | 0.7739 | 0.7051 | 0.7403 | 0.8020 | 0.5298 | 0.7533 | 0.7179 | 0.001 | 0.001 |
| KIRP-7501-01 | Proliferative | 0.6861 | 0.6648 | 0.7017 | 0.7368 | 0.6497 | 0.6530 | 0.7109 | 0.001 | 0.001 |
| KIRP-A9JO-01 | Proliferative | 0.6567 | 0.7023 | 0.7260 | 0.7455 | 0.6330 | 0.7403 | 0.7073 | 0.001 | 0.001 |
| KIRP-A5QZ-01 | Complement/Ω-oxidation | 0.7419 | 0.8232 | 0.7015 | 0.7375 | 0.7338 | 0.7229 | 0.7335 | 0.001 | 0.001 |
| KIRP-A9PQ-01 | Angiogenic | 0.6429 | 0.6417 | 0.7321 | 0.8253 | 0.6771 | 0.6929 | 0.7002 | 0.001 | 0.001 |
| KIRC-3313-01 | Proliferative | 0.7452 | 0.7181 | 0.7488 | 0.7767 | 0.5180 | 0.6977 | 0.6932 | 0.001 | 0.001 |
| KIRP-8537-01 | T-eff/Proliferative | 0.6737 | 0.7382 | 0.6365 | 0.5582 | 0.6774 | 0.6362 | 0.7256 | 0.001 | 0.001 |
| KIRC-4756-01 | Proliferative | 0.7993 | 0.6400 | 0.7280 | 0.7180 | 0.5439 | 0.7501 | 0.6979 | 0.001 | 0.001 |
| KIRP-5882-01 | T-eff/Proliferative | 0.6435 | 0.7101 | 0.6789 | 0.6128 | 0.6673 | 0.6537 | 0.7159 | 0.001 | 0.001 |
| ^†^Seven molecular subtypes were determined by non-negative factorization matrix (NMF) clustering, including NMF1 (Angio/Stroma), NMF2 (Angiogenic), NMF3 (Complement/Ω-oxidation), NMF4 (T-eff/Proliferative), NMF5 (Proliferative), NMF6 (Stromal/Proliferative) and NMF7 (snoRNA) | | | | | | | | | | |
| ^§^Distance to the template (gene signature) was estimated based on the transcriptional expression data | | | | | | | | | | |
